# Sensing Emotion in Voices: Negativity Bias and Gender Differences in a Validation Study of the Oxford Vocal (‘OxVoc’) Sounds Database

**DOI:** 10.1037/pas0000382

**Published:** 2016-09-22

**Authors:** Katherine S. Young, Christine E. Parsons, Richard T. LeBeau, Benjamin A. Tabak, Amy R. Sewart, Alan Stein, Morten L. Kringelbach, Michelle G. Craske

**Affiliations:** 1 Department of Psychology, University of California, Los Angeles and Department of Psychiatry, University of Oxford; 2 Department of Psychiatry, University of Oxford and Department of Clinical Medicine, Aarhus University; 3 Department of Psychology, University of California, Los Angeles; 4 Department of Psychiatry, University of Oxford; 5 Department of Psychiatry, University of Oxford and Department of Clinical Medicine, Aarhus University; 6 Department of Psychology, University of California, Los Angeles

**Keywords:** adult, emotional expression, infant, stimulus database, vocalization

## Abstract

Emotional expressions are an essential element of human interactions. Recent work has increasingly recognized that emotional vocalizations can color and shape interactions between individuals. Here we present data on the psychometric properties of a recently developed database of authentic nonlinguistic emotional vocalizations from human adults and infants (the Oxford Vocal ‘OxVoc’ Sounds Database; Parsons, Young, Craske, Stein, & Kringelbach, 2014). In a large sample (*n* = 562), we demonstrate that adults can reliably categorize these sounds (as ‘positive,’ ‘negative,’ or ‘sounds with no emotion’), and rate valence in these sounds consistently over time. In an extended sample (*n* = 945, including the initial *n* = 562), we also investigated a number of individual difference factors in relation to valence ratings of these vocalizations. Results demonstrated small but significant effects of (a) symptoms of depression and anxiety with more negative ratings of adult neutral vocalizations (*R*^2^ = .011 and *R*^2^ = .008, respectively) and (b) gender differences in perceived valence such that female listeners rated adult neutral vocalizations more positively and infant cry vocalizations more negatively than male listeners (*R*^2^ = .021, *R*^2^ = .010, respectively). Of note, we did not find evidence of negativity bias among other affective vocalizations or gender differences in perceived valence of adult laughter, adult cries, infant laughter, or infant neutral vocalizations. Together, these findings largely converge with factors previously shown to impact processing of emotional facial expressions, suggesting a modality-independent impact of depression, anxiety, and listener gender, particularly among vocalizations with more ambiguous valence.

Emotional expressions serve to communicate affective experiences between individuals. How these cues are interpreted can have an important impact on social interactions. For instance, appraisal theory states that emotional cues communicate an individual’s behavioral intentions, in turn guiding another’s responses (Hess, Blairy, & Kleck, 2000; Salovey & Mayer, 1990). The prominence of affective cues in social interactions is apparent from early in life with parent-infant interactions comprising largely of exchanges of emotional vocal and facial expressions (Papousek, 2007). There is a large body of work investigating responses to emotional facial expressions while comparatively little has focused on the impact of emotional vocalizations. This discrepancy is partly because there are relatively few validated databases of emotional vocalizations compared with the range of publicly available databases of affective facial stimuli.

## Emotional Vocalizations

There are two modes in which humans communicate emotion through the voice: verbal prosody and nonverbal ‘affective bursts.’ Existing research on vocal communication of emotion has largely focused on verbal prosody, whereby affect is transmitted through the intonation and melody of speech (for review, see Scherer, 2003). Stimulus databases of affective prosody typically comprise sets of highly controlled stimuli, with actors speaking semantically neutral sentences or ‘pseudosentences’ (sentences made up of nonwords, for example, the Danish Emotional Speech Database [Engberg & Hansen, 1996]; Berlin Database of Emotional Speech [Burkhardt, Paeschke, Rolfes, Sendlmeier, & Weiss, 2005]). As linguistically based stimuli, investigation of prosody is naturally limited to some extent by participants’ native language and the use of artificial (acted) stimuli.

In contrast, nonverbal affective bursts, such as laughter, crying, or screaming, have no linguistic content. Unconstrained by the mechanics of speech production, affective bursts tend to encompass a broader pitch range and characteristically different harmonic and temporal structures (Scott, Sauter, & McGettigan, 2010). Nonlinguistic affective bursts are more spontaneous expressions of affect, often accompanying intense emotional states (Scherer, 1995). As such, affective bursts are a powerful tool for investigating responses to emotion and can be used in a range of cultural settings. These bursts are also considered a more primitive mode of communication, compared with linguistic prosody, and are therefore more comparable with vocalizations of prelinguistic infants as well as the vocalizations of other species (Belin, Fecteau, & Bédard, 2004; Juslin & Laukka, 2003; Scherer, 1995).

We recently made available a set of authentic, nonlinguistic affective burst stimuli (the Oxford Vocal ‘OxVoc’ Sounds database; http://www.kringelbach.org/oxvoc/), consisting of positive, negative, and neutral vocalizations from adults and infants as well as domestic animal distress vocalizations (Parsons, Young, Craske, et al., 2014). This stimulus set complements existing databases of affective bursts generated by adult actors [for example, The Montreal Affective Voices (Belin, Fillion-Bilodeau, & Gosselin, 2008); The International Affective Digitized Sounds (Stevenson & James, 2008); Corpus of Nonverbal Vocalizations (Lima, Castro, & Scott, 2013); the Geneva Multimodal Emotion Portrayals, GEMEP (Bänziger, Mortillaro, & Scherer, 2012)]. The OxVoc database is novel in its use of authentic (nonacted) vocalizations. Unlike previous databases that contain stimuli produced by actors, the OxVoc stimuli are spontaneous, genuine expressions of emotion obtained from freely available online videos of adults in conversation or speaking directly to camera (video logs).

Authenticity in vocalizations is an important consideration as there is evidence suggesting that physical differences exist between acted and authentic emotional vocalizations (Audibert, Aubergé, & Rilliard, 2010; Jürgens, Hammerschmidt, & Fischer, 2011). Further, these differences can be detected by adult listeners, as demonstrated by variance in perceived intensity ratings and neural processing (Barker, 2013; Barkhuysen, Krahmer, & Swerts, 2007; Laukka, Neiberg, Forsell, Karlsson, & Elenius, 2011; McGettigan et al., 2015). The inclusion of infant vocalization stimuli in the OxVoc database also facilitates the investigation of how responses to emotional cues might vary with the developmental stage of the vocalizer. Similarly, including animal vocalizations permits the investigation of species-specific responses.

## Individual Differences in Emotion Perception

Early research into human emotional communication sought to demonstrate the universal nature of human emotional expressions. There is a large body of evidence supporting this notion, with findings from individuals from a range of different countries performing above chance on emotional recognition tasks both for facial expressions (e.g., Ekman & Friesen, 1971; Elfenbein & Ambady, 2002) and vocalizations (e.g., Bryant & Barrett, 2008; Koeda et al., 2013; Laukka et al., 2013; Paulmann & Uskul, 2014; Sauter, Eisner, Ekman, & Scott, 2010, 2015). More recently, research has begun to focus on what factors might impact subtle differential responding to emotional cues between individuals. The main factors investigated to date are the emotional state and gender of the listener.

### Negativity Bias in Depression and Anxiety

Altered reactivity to emotional stimuli among individuals with depression and anxiety has been consistently reported, with studies demonstrating negative bias in attention, memory and interpretation processes (for review, see Mathews & MacLeod, 2005). Negative interpretation bias is the tendency to appraise information in a negative manner (Butler & Mathews, 1983). This effect has been demonstrated in ratings of facial expressions among individuals with depression or social anxiety disorder and is particularly apparent when facial expressions are neutral or ambiguous in nature (Bourke, Douglas, & Porter, 2010; Gebhardt & Mitte, 2014; Gollan, Pane, McCloskey, & Coccaro, 2008; Joormann & Gotlib, 2006; Leppänen, Milders, Bell, Terriere, & Hietanen, 2004; Richards et al., 2002; Yoon & Zinbarg, 2008). A similar negative bias has been reported among mothers with postnatal depression rating facial expressions of their infants (Arteche et al., 2011; Stein et al., 2010). In contrast, mothers with generalized anxiety disorder (GAD) demonstrated enhanced sensitivity to happy infant facial expressions in a task requiring detection of emotion while faces ‘morph’ between different expressions. Mothers with GAD were found to detect happy expressions, but not sad expressions, at lower intensities than healthy mothers (Arteche et al., 2011).

Fewer studies have investigated interpretation bias for emotional vocalizations, with mixed findings to date. Two studies have demonstrated negative bias in the interpretation of positive and negative prosody, one in individuals with depression (Péron et al., 2011) and the other in individuals with social anxiety disorder (Quadflieg, Wendt, Mohr, Miltner, & Straube, 2007). Others have demonstrated effects specific to positive prosody in depression (Schlipf et al., 2013) or no effect for trait anxiety (Koizumi et al., 2011). Studies of infant vocalizations have not specifically investigated interpretation bias, but there have been demonstrations of overall reduced sensitivity to infant vocal distress in adults with depression. These studies have demonstrated that adults with depression have reduced abilities to discriminate pitch or ‘infant distress’ in pitch-varying infant cries, compared with healthy adults (Donovan, Leavitt, & Walsh, 1998; Young, Parsons, Stein, & Kringelbach, 2012). In addition, depressed women rated infant cries as less perceptually salient and less likely to elicit caregiving responses, compared with healthy women (Schuetze & Zeskind, 2001).

### Gender Differences

There is general empirical support for the wide-held view that females are more emotionally expressive than males (see Vigil, 2009 for review). From a theoretical perspective, it has been suggested that individual differences in expression of emotion are somewhat dictated by social and cultural norms as well as ‘display rules’ (Ekman & Friesen, 1971). A number of studies have demonstrated a female advantage in recognizing emotional facial expressions (Hall & Matsumoto, 2004; Lambrecht, Kreifelts, & Wildgruber, 2014). There is also evidence suggesting heightened physiological arousal, as well as increased attentional capture, in females compared with males in response to emotional facial expressions (Bradley, Codispoti, Sabatinelli, & Lang, 2001; Grossman & Wood, 1993), although not all studies show these effects (Whittle, Yücel, Yap, & Allen, 2011). Although not investigated extensively, there is some evidence indicating small but significant gender differences in sensitivity to emotional vocalization stimuli (as confirmed by meta-analysis; Bradley et al., 2001). These studies have typically demonstrated greater accuracy of emotion identification among vocal cues in females compared with males (Belin et al., 2008; Bonebright, Thompson, & Leger, 1996; Thompson & Voyer, 2014), although other studies have demonstrated no such gender differences (Lima, Alves, Scott, & Castro, 2014; Raithel & Hielscher-Fastabend, 2004), including a previous study conducted using the OxVoc database in a sample of 34 adults (Parsons, Young, Craske, et al., 2014).

Previous work has also demonstrated interaction effects between the gender of the listener and the gender of the individual producing the vocalization (the vocalizer gender). In one study, higher accuracy of emotion identification was reported for female participants listening to female vocalizations than either males listening to females or females listening to males and lower accuracy still for males listening to male vocalizations (Belin et al., 2008). Complex interaction effects have also been reported for the perception of authenticity in vocalizations. In one study examining laughter, males tended to rate both authentic and simulated female laughter as genuine, whereas women tended to rate both types of female laughter as simulated, with no gender differences observed in perceived authenticity in male laughter (McKeown, Sneddon, & Curran, 2015). Among infant vocalizations, there is mixed evidence for gender differences. For infant crying, prior work has demonstrated similar levels of ‘perceived distress’ and ‘desire to respond’ in females and males (Donate-Bartfield & Passman, 1985; Leger, Thompson, Merritt, & Benz, 1996). Studies of physiological reactivity, however, suggest gender differences in heart rate reactivity while listening to infant cries (although the direction of this effect varies across studies; Brewster, Nelson, McCanne, & Lucas, & Milner, 1998; Furedy et al., 1989; Out, Pieper, Bakermans-Kranenburg, & Van Ijzendoorn, 2010). Other work has failed to demonstrate robust gender differences for the effect of infant vocalizations on subsequent motor responses (Parsons, Young, Parsons, Stein, & Kringelbach, 2012; Young, Parsons, Stein, & Kringelbach, 2015) or early neural processing (Parsons, Young, Joensson, et al., 2014; Parsons et al., 2012; Young et al., 2016).

## Aims and Hypotheses

The current study had two primary aims. The first was to assess the test–retest reliability of perceived valence and validity of affective categorization of emotional vocal stimuli in the OxVoc database in a large sample of young adults. The valence dimension was selected based on our previous work demonstrating consistency between perceived valence and experimenter defined stimulus categories (Parsons et al., 2014). The second aim was to investigate the impact of key individual differences (symptoms of anxiety and depression and listener gender) on ratings of perceived valence of adult and infant emotional vocalizations. This study aims to comprehensively investigate these individual difference factors in relation to a large stimulus set of human affective vocalizations from both adults and infants. Existing studies of individual differences in the perception of emotional expressions have typically been small in size. Here, we investigate individual difference factors in a large group (*N* = 945) to provide more reliable estimates of effects. We hypothesized that (a) higher levels of depression or anxiety symptoms would be associated with more negative ratings of emotional vocalizations and (b) females would rate emotional vocal expressions more extremely than males.

## Method

### Participants

Participants were recruited using online advertising from the University of California, Los Angeles (UCLA) Psychology Department Subject Pool of undergraduate students who received course credit for completing the study. Ethical approval was granted by the UCLA institutional review board and written informed consent was obtained from all participants. In total, 995 participants were recruited. Twenty-eight participants were excluded for incomplete ratings data, 11 for incomplete questionnaire data, nine who reported being a parent, and two with self-reported hearing problems, leaving a total sample of 945 participants.

### Self-Report Measures

Participants provided basic demographic information regarding age, gender, and ethnicity on an online form (administered via Qualtrics, Provo, UT). Participants were young adults (age: *M* = 20.25 years, *SD* = 2.71) and predominantly female (752 female, 193 male). Participants self-identified as Asian (*n* = 427), Caucasian (*n* = 223), Hispanic/Latino (*n* = 143), and a range of other racial/ethnic backgrounds (*n* = 152).

As our goal was to investigate the nature of negative bias across the anxiety and depression spectrums, we used a dimensional assessment of symptoms, rather than grouping of individuals based on categorical diagnosis (Cuthbert & Insel, 2013; Mathews & MacLeod, 2005). Symptoms of depression were assessed using the Edinburgh Postnatal Depression Scale (EPDS; a measure with high specificity and sensitivity; Cox, Chapman, Murray, & Jones, 1996; Cox, Holden, & Sagovsky, 1987). This 10-item measure was originally developed as a screening tool for postnatal depression, but has since been validated and used in studies with men and women outside the postnatal period (termed the ‘EDS’; Matthey, Barnett, Kavanagh, & Howie, 2001). Here we used a modified 9-item version, with the suicidality item removed. Alternative versions of the EDS excluding the suicidality item (EDS-5; Eberhard-Gran, Eskild, Samuelsen, & Tambs, 2007) have previously shown this item does not substantially contribute to explaining variance of the full scale. The majority of participants (*n* = 775, 82%) scored below the cut-score on this measure (*M* = 8.50, *SD* = 4.39, see Table 1; cut-off >12, modified to account for item removal). The Generalized Anxiety Disorder Questionnaire-IV (GAD-Q-IV) was used for assessing symptoms of anxiety. It is a 9-item self-report measure with high sensitivity and specificity for detecting symptoms of generalized anxiety disorder (GAD), based on the *Diagnostic and Statistical Manual of Mental Disorders*, fourth edition (*DSM–IV*) criteria (Newman et al., 2002). The majority of participants (*n* = 836, 88%) scored below cut-off on this measure (cut-off >9.4, *M* = 5.22, *SD* = 3.14, see Table 1).[Table-anchor tbl1]

### Stimuli

All 173 sound stimuli from the Oxford Vocal (OxVoc) Sounds database (Parsons, Young, Craske, et al., 2014) were used in this study. This database contains affective vocal sound stimuli from human adults and infants and domestic animals. All stimuli are genuine (nonacted) vocalizations obtained from a variety of sources. In brief, infant vocalizations were obtained from videos of infants interacting with their caregivers in their own homes. A comparable set of adult vocalizations was obtained from freely available online sources (primarily online video logs) aiming to match the infant stimuli in the range of vocal affect and number of exemplars. Domestic animal vocalizations were obtained from freely available online sources (see Parsons, Young, Craske, et al., 2014 for more details). From all recordings, stimuli were selected based on three primary criteria: (a) sounds were free from background noise, (b) sounds contained no linguistic content, and (c) vocal bursts lasted 1 to 2 s in duration. All stimuli obtained that met these criteria were included in the database.

Using visual and contextual information from source videos, extracted stimuli were separated into categories of positive, negative, and neutral valence (only negative valence was available for domestic animal vocalizations). Categorization was performed by two authors (KSY, CEP) and disagreements were resolved through discussion. The database consists of the following vocalization categories: adult cries (negative, 19 female), adult neutral sounds (15 male, 15 female), adult laughs (positive, 15 male, 15 female), infant cries (negative, *n* = 19), infant neutral sounds (*n* = 30), infant laugh sounds (positive, *n* = 18), domestic animal sounds (negative, *n* = 30). Adult neutral sounds consisted of ‘uhm’ and ‘ehm’ sounds, occurring during gaps in speech. Infant neutral sounds consisted of ‘babbling’ sounds (e.g., ‘bah’, ‘dah’ etc.), containing no specific affect. We were unable to attain any exemplars of adult male cry vocalizations from available online sources. The present analysis focuses on the human vocalizations, but information on ratings and categorization of domestic animal sounds are presented in the Supplementary materials (test–retest reliability of perceived valence and validity of affective categorization results, Table S1).

### Procedures

All participants completed valence ratings for all stimuli from the OxVoc database. After presentation of each stimulus, participants completed ratings on a vertical visual analogue scale (VAS; encoded on a continuous numerical scale, with a sensitivity of two decimal places from + 4.00 ‘very positive’ to −4.00 ‘very negative’). Participants then completed the self-report measures described above. A subset of participants (*n* = 562) completed a second set of ratings of the same stimuli 20 min later as a measure of test–retest reliability of perceived valence, similar to procedures for validation of a facial expressions database (Tottenham et al., 2009). The same participants also completed a categorization task to assess the validity of experimenter-defined stimulus categories (as in Belin et al., 2008; Tottenham et al., 2009). In the categorization task, participants listened to each stimulus and then categorized it as ‘cry/distressed,’ ‘laugh,’ ‘sound with no emotion’ (i.e., neutral), or ‘could not categorize’ (because of technical errors with six participants the final sample size for this task was *n* = 556).

The order of stimulus presentation was randomized between each phase of the task and for each participant. Following an introductory phase, during which participants practiced the rating procedure, the task was fully automated. Participants had a maximum of five seconds to provide a response to each stimulus, after which they were automatically moved on to the next trial. The experiment was programmed in Presentation software (www.neurobs.com). Participants made responses using the ‘UP’ and ‘DOWN’ arrows on the keyboard for the VAS ratings, and keys 1 to 4 for the categorization task. Auditory stimuli were presented through headphones at a volume that was comfortable for each participant. In total, this procedure took between 50 and 60 min. Up to four participants completed the study concurrently at different workstations in the same laboratory under the supervision of a research assistant.

### Statistical Analyses

For the first aim of examining psychometric properties of perceived affect, intraclass correlations were performed to assess interparticipant reliability in perceived valence across stimuli. Pearson’s correlation coefficients were computed to investigate reliability of perceived valence over time. Chance-corrected accuracy scores (Cohen’s kappa) were used to assess validity of the existing experimenter-determined categorization of stimuli into affective groups.

For the second aim of testing the role of individual differences, hierarchical linear regression analyses were performed to investigate the relationship between symptoms of depression and anxiety and listener gender on valence ratings of adult and infant vocalizations. Analyses were performed separately for each category of stimulus (adult cry, adult laugh, adult neutral, infant cry, infant laugh and infant neutral vocalizations). Outliers for all major study variables were defined as scores falling above the 75th percentile plus 1.5 times the interquartile range, or falling lower than the 25th percentile minus 1.5 times the interquartile range and were removed from analyses. All analyses were conducted using SPSS version 23. Correction for multiple comparisons was implemented using a false discovery rate (FDR) corrected significance level for 36 tests resulting in statistical significance at the level of *p* < .0043 (Benjamini & Hochberg, 1995).

## Results

### Interparticipant Reliability of Perceived Valence

Intraclass correlations were computed to assess the extent of interrater agreement on ratings of valence across stimuli for both the first and the second rating period (encoded on a continuous scale from −4.00 ‘very negative’ to + 4.00 ‘very positive’). For both rating periods, interparticipant agreement in valence ratings across all stimuli was high (first period, ICC(2,562) = .99, *p* < .001; second period, ICC(2,562) = .99, *p* < .001). Examination of interrater agreement in valence ratings for individual categories of stimuli was also shown to be high (all ICC > .98; ratings for individual subcategories are presented in Table 2).[Table-anchor tbl2]

### Test–Retest Reliability of Perceived Valence

Valence ratings across all sounds were highly correlated from time one to time two, *r* = .82, *n* = 562, *p* < .001. Ratings of individual subcategories of sounds were also all highly correlated (*p* < .001), with all *r*s > .80, apart from repeat ratings of adult neutral vocalizations which were correlated at *r* = .72 (see Table 2).

### Validity of Affective Categorization

**Figure 1 fig1:**
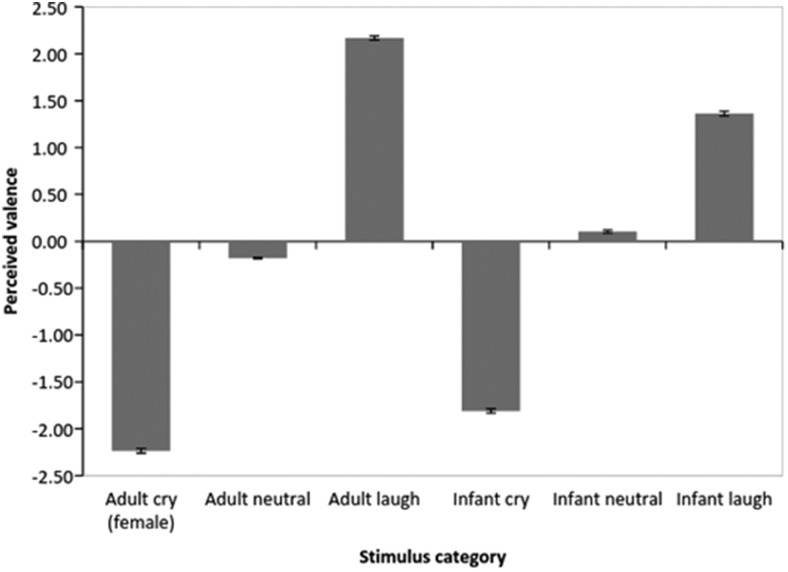
Mean ratings of perceived valence across stimulus categories demonstrated initial validity of categorization with negative stimuli rated negatively, neutral stimuli rated somewhat neutrally, and positive stimuli rated positively. Error bars represent mean ± standard error.

**Figure 2 fig2:**
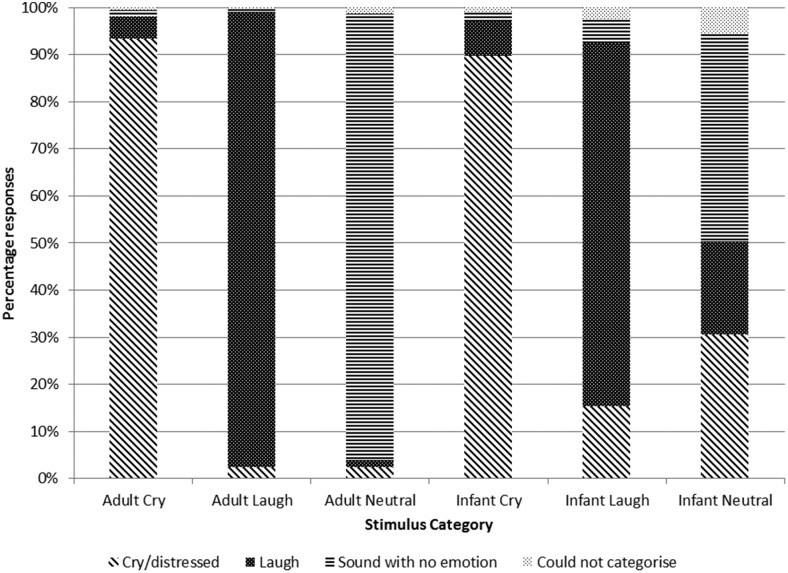
Results of the categorization task across stimulus categories. Overall, accuracy was high for the adult vocalizations and slightly lower for the infant vocalizations. Categorization accuracy was lowest for the infant neutral vocalizations.

Examining valence ratings across categories demonstrated that adult female cries and infant cries were rated negatively, adult and infant neutral vocalizations were rated neutrally, and that adult and infant laughter stimuli were rated positively (see Figure 1, consistent with findings in Parsons et al., 2014). Categorization of the adult vocalizations (cry, laughter and neutral) had high validity, with chance-corrected accuracy scores (Cohen’s Kappa) above 90% and small (<.10) standard deviations (see Figure 2). The infant cry vocalizations were also accurately categorized (>80%), while scores for infant laugh stimuli were lower (70%) and scores for neutral vocalizations were below chance (26%). Examination of the data at the stimulus level revealed that 10 of the infant neutral stimuli were correctly categorized (at >50% accuracy level), five were perceived by more than 50% of participants as cry/distressed sounds, and the remaining nine had no clear overall categorization. Of note, the proportion of individuals selecting the ‘could not categorize’ option was very low (mean proportion across all stimuli = .02).[Fig-anchor fig1][Fig-anchor fig2]

### Individual Difference Analyses

Hierarchical linear regression analyses were conducted to investigate individual differences in perceived valence of adult and infant vocalizations. There was a strong correlation between scores on the depression (EPDS) and anxiety (GADQ) scales, *r* = .66, *p* < .001, so analyses were performed separately for these measures to avoid confounds of multicollinearity. First, we examined the main effect of depression symptoms on valence ratings across categories of stimulus. We observed a significant negative relationship between EPDS score and ratings of adult neutral vocalizations (*b* = −.006, *SE* = .002, *p* = .002, *R*^2^ = .011). There was no significant effect of EPDS score on ratings of other vocalizations (all *p*s > .05, see Table 3). The main effect of anxiety symptoms on valence ratings demonstrated similar results. There was a significant negative relationship between GADQ score and ratings of adult neutral vocalizations (*b* = −.007, *SE* = .003, *p* = .006, *R*^2^ = .008), with no significant effects for all other vocalization categories (all *p*’s > .05, see Table 3).[Table-anchor tbl3]

We also examined the main effect of listener gender (coded as female = 0, male = 1) on ratings of emotional vocalizations, after statistically controlling for the effect of EPDS or GADQ score. In both sets of analyses, we observed significant effects of listener gender on valence ratings of adult neutral vocalizations (controlling for EPDS score: *b* = .061, *SE* = .021, *p* = .003, *R*^2^ = .021; controlling for GADQ score: *b* = .060, *SE* = .021, *p* = .004, *R*^2^ = .018), and infant cry vocalizations (controlling for EPDS score: *b* = −.166, *SE* = .058, *p* = .004, *R*^2^ = .010; controlling for GADQ score: *b* = −.168, *SE* = .058, *p* = .004, *R*^2^ = .011). Compared with males, females rated adult neutral vocalizations less negatively (male: *M* = −.26, *SD* = .73; female: *M* = −.20, *SD* = .76) and infant cry vocalizations more negatively (male: *M* = −1.65, *SD* = .35; female: *M* = −1.83, *SD* = .34). There was no significant effect of gender on ratings of other vocalizations (all *p*’s > .05, see Table 3).

We then examined the interaction between depression symptoms and listener gender on ratings of valence across all categories of adult and infant vocalizations and found no significant effects (all *p*s > .05, see Table 3). Similarly, there were no significant interaction effects of anxiety symptoms and listener gender on valence ratings (all *p*s > .05, see Table 3).

To investigate the effect of vocalizer gender, analyses reported above were repeated separately for adult male and female neutral and positive vocalizations. Main effects of depression symptoms were observed for both male and female neutral vocalizations (male: *b* = −.008, *SE* = .002, *p* = .001, *R*^2^ = .011; female: *b* = −.006, *SE* = .002, *p* = .004, *R*^2^ = .009). Main effects of anxiety symptoms did not meet significance (all *p*s > .01, see Table S2). There was a significant main effect of listener gender on female neutral, but not male neutral vocalizations (female: *b* = .068, *SE* = .021, *p* = .001, *R*^2^ = .019; male: *b* = .015, *SE* = .026, *p* = .571, *R*^2^ = .004). Female listeners rated female neutral vocalizations less negatively (*M* = −.06, *SD* = .76) than male listeners (*M* = −.15, *SD* = .38; *t*(873) = −2.95, *p* = .003, *d* = 3.97). Main effects of depression symptoms, anxiety symptoms and listener gender were not significant for all other vocalization categories (see Supplementary Materials for full details, Table S2). Results demonstrated no significant interaction effects of depression symptoms and listener gender or anxiety symptoms and listener gender on male or female neutral or positive vocalizations (all *p*’s > .09, Table S2).

## Discussion

We demonstrate that adults correctly categorize and consistently rate the valence of nonlinguistic emotional vocalizations in the OxVoc Sounds Database. Reliability of perceived valence ratings and validity of affective categorization was demonstrated to be comparable with that of other widely used databases of emotional expressions (both vocalizations; Lima et al., 2013; and facial expressions; Tottenham et al., 2009) in all categories of stimuli, except infant neutral vocalizations. Considering individual differences in ratings of emotional vocalizations, we found a small but significant effect of depression and anxiety symptoms on ratings of adult neutral vocalizations such that greater symptom levels were associated with more negative ratings. There were no such effects of depression/anxiety symptoms on perceived valence of adult laughter or cry vocalizations, or any of the infant vocalization categories. We also found small effects of gender, such that females rated adult neutral vocalizations less negatively and infant cry vocalizations more negatively than males did. There were no significant gender differences in ratings of other categories of stimuli.

### Psychometric Properties

In a large sample of young adults, we demonstrated high agreement between listeners in their perceived valence of emotional vocalizations from adults and infants in the OxVoc database. We also found strong test–retest reliability in ratings of perceived valence in a subgroup of participants who provided two sets of valence ratings for the same stimuli. The category of stimuli with the lowest consistency in test–retest analyses was the adult neutral vocalizations (*r* = .72, whereas all other categories *r* > .80). Relatively greater variability in perceived valence among these stimuli might be related to their inherent ambiguity, as compared with extreme positive (laughter) and extreme negative (crying) vocalizations. A similar effect has been noted in relation to the ambiguity of neutral facial expressions (Beevers, Wells, Ellis, & Fischer, 2009; Richards et al., 2002; Yoon & Zinbarg, 2008). Nonetheless, levels of interrater reliability in perceived valence ratings across all categories were comparable to those for other stimulus databases of human emotional expressions.

The validity of affective categorization was found to be high for all stimulus categories except infant neutral vocalizations. A substantial proportion of these stimuli were found to be more commonly perceived as negative (‘crying’), rather than neutral (‘babbling’) sounds. Analyses reported here include all stimuli available in the database to provide a comprehensive overview, demonstrating a variety of stimuli including ambiguous and hard-to-categorize cues. Future studies may wish to select subgroups of stimuli (e.g., particularly unambiguous stimuli) depending on individual research goals. Details of stimulus-level analyses are provided in Supplementary Materials (Table S3) for this purpose.

### Symptoms of Depression and Anxiety

The association between higher levels of self-reported symptoms of depression and anxiety and more negative ratings of adult neutral vocalizations is consistent with previous reports of negativity bias of neutral facial expressions in individuals with depression (Gollan et al., 2008; Joormann & Gotlib, 2006; Leppänen et al., 2004) and social anxiety disorder (Richards et al., 2002; Yoon & Zinbarg, 2008). Here, we found no evidence for a differential impact of depression or anxiety symptoms. The specificity of negative bias demonstrated here to adult neutral vocalizations is consistent with previous work suggesting a stronger negative bias among more ‘ambiguous’ stimuli (Beevers et al., 2009). The positive and negative stimuli used here (laughter and cries) are by their nature relatively unambiguous, as demonstrated by the high test–retest reliability of perceived valence ratings and high categorization accuracy. Neutral vocalization stimuli were found to be more ambiguous as demonstrated by lower correlations in test-retest analyses. These findings suggest that observed negative interpretation bias associated with depression and anxiety extend to ambiguous adult nonlinguistic vocalizations, even among a nonclinical sample. Of note, there was no observed effect of depression and anxiety symptoms on interpretation of infant vocalizations, as measured by valence ratings and categorization. Previous reports have suggested a reduced sensitivity to distress in infant vocalizations among individuals with depression, as indexed by reduced accuracy in pitch and distress discrimination of these sounds as well as lower ratings of salience (Donovan et al., 1998; Schuetze & Zeskind, 2001; Young et al., 2012). The lack of significant effects here suggests that the impact of depression on responses to infant cues might be limited to samples with more severe symptom levels, samples of older individuals or samples of parents.

### Gender Differences

Previous literature has reported heightened sensitivity to emotional cues among females compared with males (Belin et al., 2008; Hall, 1978; Hall & Matsumoto, 2004; Lambrecht et al., 2014). Here we demonstrated that male listeners rated adult neutral vocalizations more negatively than female listeners, an effect primarily driven by ratings of female neutral vocalizations. These effects in a large sample of young adults suggest a specific gender difference, rather than a global difference in emotional reactivity. We hypothesized that females would have more extreme ratings than males, but findings showed that males provided more negative ratings of neutral vocalizations than females. The reason for this effect requires further investigation, but it complements existing findings demonstrating interaction effects of listener gender and vocalizer gender in perceived valence (Belin et al., 2008; McKeown et al., 2015). As noted earlier, findings on gender differences in the perception of emotion in voices have been mixed, perhaps related to power issues. In previous work, using a small sample size (*n* = 34), we did not observe gender differences in valence ratings, whereas in the current study using a large sample size (*n* = 945), we were able to detect small but significant effects of gender.

Female listeners also rated infant cry vocalizations more negatively than male listeners. This finding might be interpreted as a specific example of heightened emotional reactivity among female, compared with male listeners. Another possible explanation is the potential for greater experience with infant caregiving in females compared with males (even in a nonparent sample), perhaps impacting on the extent of perceived negative valence. Investigation of changes in ratings of infant cues would be an important avenue for future research not only for improving understanding of individual differences in perceived valence, but more broadly for investigating how sensitivity to infant cues might be altered by the experience of caregiving. It is also possible that such gender differences are limited to self-reported emotional reactivity while studies investigating other aspects of responses to infant cries (e.g., psychophysiological) have failed to demonstrate robust gender-related effects (Parsons, Young, Bhandari, et al., 2014; Parsons et al., 2012; Young et al., 2012).

### Strengths and Limitations

Strengths of this study include the large sample size recruited, as well as the large set of stimuli used ranging across different categories of emotion. High inter- and intraparticipant reliability of perceived valence ratings and high validity of affective categorization of stimuli were observed. In addition, the majority of previous studies investigating negative bias have demonstrated the effect among smaller samples of individuals with a full clinical diagnosis. Here we demonstrate a similar effect in a large nonclinical sample, albeit including a broad range of self-reported symptomatology. It should be noted that although effects reported here met statistical significance, effect sizes were small, highlighting the importance of investigating other factors affecting perceived valence in emotional vocalizations as well as future use of large sample sizes to demonstrate such effects. In addition, our test–retest period was relatively short (20 min) and individual consistency in perceived affect among vocal stimuli might helpfully be assessed over longer time periods.

Future work might also consider other dimensions in emotional stimuli, particularly perceived arousal and the impact of other contextual cues (e.g., facial expressions). Inclusion of negative male vocalization stimuli (male cries) would also allow further investigation of vocalizer gender differences across categories of affective stimuli. Additionally, we describe our adult stimuli as authentic in nature because stimuli were obtained from online recordings where the intention of the recording was not to generate emotional vocalizations. However, it is possible that mere knowledge of being recorded (i.e., observation bias) influenced the type, range and quality of vocalizations produced.

Participants in this study were all undergraduate students and predominantly female, limiting the generalizability of the findings. Nonetheless, despite the homogeneity of the sample in terms of age and education, there was substantial ethnic heterogeneity and variability within measures across the sample. Although a large body of work has investigated the impact of an individual’s race and ethnicity on emotional reactivity, this was not a factor investigated in the current study. The UCLA undergraduate population has a range of racial and ethnic backgrounds, but a high proportion of individuals are first- or second-generation Americans. We considered our self-report measure of race and ethnicity to be insufficient to quantify acculturation within this group, a factor that likely varies widely in our sample within individuals reporting the same race/ethnicity. Future investigation of these factors might be of particular interest in studying the impact of cultural effects on perceived emotion in vocalizations.

### Future Applications

As a validated set of emotional vocal stimuli, it is hoped that this stimulus set may be useful across a range of studies aiming to investigate emotional perception and processing. Psychometric data provide normative ratings against which responses from groups of individuals with specific impairments or disorders might be compared. The inclusion of both infant and adult vocalizations from basic affective categories (positive, negative, neutral) permits initial assessment of developmental aspects of emotional processing. Future addition of other types of affective vocalizations (e.g., disgust, surprise and anger) would helpfully extend this database, as would examples of vocalizations from individuals of other ages (e.g., children, adolescents, older adults). Finally, a large number of exemplars of each stimulus category were included for use in neuroimaging contexts (particularly electroencephalography and magnetoencephalography) where large numbers of unique stimuli are often required.

### Conclusions

In summary, we demonstrated that the stimuli within the OxVoc database have high reliability of perceived valence and affective categorization validity, making the database of potential use for future studies investigating vocal emotional processing. We also present findings of individual differences in perceived valence of nonlinguistic emotional vocalizations. Among a large sample, we demonstrated effects of depression and anxiety symptoms and participant gender on valence ratings of adult neutral vocalizations specifically. The observed effects of depression and anxiety are comparable with previous findings of processing of facial expressions in clinical populations, suggestive of a modality-independent bias that is dimensional in nature. Observed gender differences were limited to ratings of female neutral and infant cry vocalizations, contrary to the notion of global gender differences in emotional reactivity.

## Supplementary Material

10.1037/pas0000382.supp

## Figures and Tables

**Table 1 tbl1:** Gender Ratios Among Individuals Scoring Above and Below the Cut-Scores for Depression (Measured Using The EPDS) and Anxiety (GADQ)

Subject	EPDS > 12	EPDS < 12	GADQ > 9.4	GADQ < 9.4	Total
Male	27 (14.0%)	166 (86.0%)	18 (9.3%)	175 (90.7%)	193
Female	143 (19.0%)	609 (81.0%)	91 (12.1%)	661 (87.9%)	752
Total	140	805	109	836	945

**Table 2 tbl2:** Inter-Participant Agreement (Intra-Class Correlations [ICC]), Test–Retest Reliability (Pearson’s r), and Chance-Corrected Accuracy Scores (Cohen’s Kappa) Averaged Across Stimulus Subcategories

Subcategory	Stimuli (*n*)	ICC time 1	ICC time 2	Pearson’s (*r*)	Cohen’s kappa *M* (*SD*)
Adult cry	19	.99	.98	.85	.91 (.08)
Adult laugh	30	.99	.99	.88	.96 (.05)
Adult neutral	30	.99	.99	.72	.93 (.03)
Infant cry	21	.99	.99	.84	.86 (.15)
Infant laugh	18	.99	.98	.83	.70 (.14)
Infant neutral	25	.99	.99	.80	.26 (.22)

**Table 3 tbl3:** Results of Hierarchical Linear Regressions, Depression or Anxiety, and Listener Gender Entered at the First Level and the Interaction Term Entered on the Second Level

Variable	*n*	*b*	*SE*	*p*	*R*^2^	*R*^2^ change
Main effect of depression symptoms						
Adult cry	929	−.008	.006	.195	.002	
Adult laugh	918	−.001	.005	.905	.000	
Adult neutral^a^	887	−.006	.002	.002	.011	
Infant cry	929	−.007	.006	.234	.002	
Infant laugh	923	−.001	.006	.813	.000	
Infant neutral	912	−.008	.004	.043	.004	
Main effect of anxiety symptoms						
Adult cry	937	−.017	.008	.031	.005	
Adult laugh	926	.005	.007	.475	.001	
Adult neutral^a^	895	−.007	.003	.006	.008	
Infant cry	937	−.010	.007	.161	.002	
Infant laugh	931	.005	.008	.528	.000	
Infant neutral	920	−.003	.005	.611	.000	
Main effect of listener gender^b^						
Adult cry	929	−.089	.061	.144	.004	
Adult laugh	918	.118	.054	.028	.005	
Adult neutral^a^	887	.061	.021	.003	.021	
Infant cry^a^	929	−.166	.058	.004	.010	
Infant laugh	923	.135	.064	.037	.005	
Infant neutral	912	.044	.042	.289	.006	
Interaction effect of depression symptoms and listener gender						
Adult cry	929	−.009	.015	.545	.005	.000
Adult laugh	918	−.005	.013	.700	.005	.000
Adult neutral	887	−.005	.005	.322	.022	.001
Infant cry	929	−.003	.014	.808	.010	.000
Infant laugh	923	−.005	.015	.761	.005	.000
Infant neutral	912	−.002	.010	.830	.006	.000
Interaction effect of anxiety symptoms and listener gender						
Adult cry	937	.008	.019	.684	.007	.000
Adult laugh	926	−.007	.017	.671	.006	.000
Adult neutral	895	−.005	.007	.434	.018	.001
Infant cry	937	−.009	.018	.623	.011	.000
Infant laugh	931	.006	.020	.781	.005	.000
Infant neutral	920	−.013	.013	.321	.002	.001
^a^ Significant effect (*p* < .0043, false discovery rate correction for multiple comparisons). ^b^ Effect after statistically controlling for depression symptoms (results were the same when statistically controlling for anxiety symptoms).
