# Supplementary material for: Sensing Emotion in Voices: Negativity Bias and Gender Differences in a Validation Study of the Oxford Vocal (‘OxVoc’) Sounds Database
Source: Psychol Assess. 2016 Sep 22;29(8):967–77. doi: 10.1037/pas0000382 (PMC5362357; doi:10.1037/pas0000382)
Supplement: Supplementary file 2 [file z1t009163143so2.docx]

**Supplemental Materials**

**Sensing Emotion in Voices: Negativity Bias and Gender Differences in a Validation Study of the Oxford Vocal ('Oxvoc') Sounds Database**

**by K. S. Young et al., 2016, *Psychological Assessment***

**http://dx.doi.org/10.1037/pas0000382**

Table S1. Reliability and validity statistics for animal vocalisation stimuli

|  | Stimuli (n) | ICC time 1 | ICC time 2 | Pearson’s (*r*) | Cohen’s Kappa, *M*(SD) |
| --- | --- | --- | --- | --- | --- |
| Cat ‘meows’ | 15 | .96 | .98 | .98 | .62 (.11) |
| Dog ‘whines’ | 15 | .92 | .97 | .93 | .86 (.07) |

Table S2. Statistical results of analyses investigating differences in vocaliser gender on the association of depression and anxiety symptoms and listener gender on ratings of adult emotional vocalisations

|  | *n* | b | SE | p | R^2^ | R^2^ change |
| --- | --- | --- | --- | --- | --- | --- |
| ***Main effect of depression symptoms*** | | | | | | |
| Male laugh | 920 | .002 | .006 | .744 | .000 |  |
| Female laugh | 924 | -.001 | .005 | .865 | .000 |  |
| Male neutral* | 899 | -.008 | .002 | .001 | .011 |  |
| Female neutral* | 867 | -.006 | .002 | .004 | .009 |  |
| ***Main effect of anxiety symptoms*** | | | | | | |
| Male laugh | 928 | .009 | .007 | .238 | .002 |  |
| Female laugh | 932 | .003 | .007 | .624 | .000 |  |
| Male neutral | 907 | -.006 | .003 | .064 | .004 |  |
| Female neutral | 875 | -.007 | .003 | .010 | .008 |  |
| ***Main effect of listener gender^a^*** | | | | | | |
| Male laugh | 920 | .109 | .058 | .062 | .004 |  |
| Female laugh | 924 | .079 | .054 | .148 | .002 |  |
| Male neutral | 899 | .019 | .026 | .469 | .012 |  |
| Female neutral* | 867 | .069 | .021 | .001 | .021 |  |
| ***Interaction effect of depression and listener gender*** | | | | | | |
| Male laugh | 920 | -.010 | .014 | .463 | .004 | .001 |
| Female laugh | 924 | -.003 | .013 | .821 | .002 | .000 |
| Male neutral | 899 | -.010 | .006 | .092 | .015 | .003 |
| Female neutral | 867 | .000 | .005 | .963 | .021 | .000 |
| ***Interaction effect of anxiety and listener gender*** | | | | | | |
| Male laugh | 928 | -.015 | .018 | .414 | .006 | .001 |
| Female laugh | 932 | .006 | .017 | .701 | .003 | .000 |
| Male neutral | 907 | -.013 | .008 | .097 | .007 | .003 |
| Female neutral | 875 | -.003 | .007 | .629 | .020 | .000 |

*indicates significance at *p* < .0075 (false discovery rate correction for 20 comparisons), ^a^ after statistically controlling for depression symptoms (results were the same when controlling for listener anxiety).
